# Resident Memory T Cells (T_RM_) Are Abundant in Human Lung: Diversity, Function, and Antigen Specificity

**DOI:** 10.1371/journal.pone.0016245

**Published:** 2011-01-26

**Authors:** Rahul Purwar, James Campbell, George Murphy, William G. Richards, Rachael A. Clark, Thomas S. Kupper

**Affiliations:** 1 Department of Dermatology, Brigham and Women's Hospital, Harvard Skin Disease Research Center, Boston, Massachusetts, United States of America; 2 Department of Pathology, Brigham and Women's Hospital, Harvard Medical School, Boston, Massachusetts, United States of America; 3 Tissue Bank, Brigham and Women's Hospital, Boston, Massachusetts, United States of America; University of Leuven, Rega Institute, Belgium

## Abstract

Recent studies have shown that tissue resident memory T cells (T_RM_) are critical to antiviral host defense in peripheral tissues. This new appreciation of T_RM_ that reside in epithelial tissues and mediate host defense has been studied most extensively in skin: adult human skin contains large numbers of functional T_RM_ that express skin specific markers. Indeed, more than twice as many T cells reside in skin as in peripheral blood. This T cell population has a diverse T cell receptor repertoire, and can produce a broad array of cytokines. More recently, we have begun to examine other epithelial tissues for the presence of resident T cells. In the present study, we asked whether analogous populations of resident T cells could be found in human lung. We were able to demonstrate abundant resident T cells in human lung-more than 10 billion T cells were present. Lung T cells were largely of the effector memory T cell (T_EM_) phenotype, though small numbers of central memory T cells (T_CM_) and T regulatory cells (T_reg_) could be identified. Lung T cells had a diverse T cell receptor repertoire and subsets produced IL-17, IL-4, IFNγ, as well as TNFα. A significant number of lung T_RM_ CD4+Th cells produced more than one cytokine, identifying them as “multifunctional” Th1 type cells. Finally, lung T_RM_, but not T_RM_ resident to skin or T cells from blood, proliferated in response to influenza virus. This work suggests that normal human lung contains large numbers of T_RM_ cells, and these cells are poised to respond to recall antigens previously encountered through lung mucosa. This population of T cells may contribute to the pathogenesis of asthma and other T cell mediated lung diseases.

## Introduction

Until very recently, T cell memory was thought to reside primarily in blood and lymphoid tissues, sites from which effector T cells could be recruited when needed for primary or secondary immune responses in peripheral tissues. T cells were thought to be rare in peripheral tissues [1], [2], [3]. Several lines of evidence have emerged recently that require revision of this view. Recently, we demonstrate that large numbers of resident T cells (T_RM_) can be identified in, and isolated from, normal non-inflamed human skin [4], [5], [6], [7]. These analyses have led to the startling observation that there are roughly 20 billion T cells in the skin of an adult human, twice the total number of T cells in blood. More than 50 times more skin homing effector memory T cells (T_EM_) reside in skin as compared to blood. These skin resident T cells are highly diverse by Tcr Vβ spectratyping, and include populations of CD4 and CD8 positive cells capable of producing different combinations of cytokines, including TNFα, IL-17, IFNγ, IL-13, and IL-4. In parallel, a series of mouse models have demonstrated that skin homing T_EM_ are generated from naïve T cells after antigen encounter in lymph nodes draining skin, and these T_EM_ rapidly migrate to and extravasate in skin [8]. These recruited T cells remain in skin for long periods of time, and can mediate recall immune memory responses many months after their initial recruitment. Recent studies have demonstrated that protective T cell responses to viral infections in skin or lung were largely mediated by these T_RM_ cells, rather than T cells recruited from blood or secondary lymphoid tissues [9], [10], [11], [12]. Taken together, these observations have led to a paradigm shift in the concept of T cell memory in skin, and suggest that host defense to pathogens previously encountered through skin is mediated to a significant degree by tissue T_RM_.

While extrapolation of these findings to other peripheral epithelial tissues that interface with the environment is a logical next step, studying these tissue resident T cells in other tissues has not been straightforward. Lung is critical epithelial interface with the environment, and T cells are critically important for the host defense of this organ as well. Histological examination of normal human skin, and counting of CD3 positive cells in histological sections, revealed unexpectedly large numbers of T cells, such that it was extrapolated that 2×10^10^ T cells resided in human skin. In the present study, we applied the same approach to sections of histologically uninflamed human lung, counting CD3 positive cells in lung parenchyma. Extrapolations from those data suggest that human lungs contain roughly 1×10^10^ T cells, a number comparable to the number of T cells in human blood. Thus, by histology alone, it was possible to identify unexpectedly large numbers of T cells in human lung.

The results alluded to above in human skin were enabled by the development of a novel explant method in our lab that markedly increases the yield of recovery of T cells without either activation or alteration of phenotype. We asked if the same method could be applied to lung with similar results. In the present study, we compare recovery of lung T_RM_ cells by conventional enzymatic digestion to a novel explant method first developed for isolation of skin T_RM_ cells [5]. We find that at least 10 fold more T cells can be isolated from lung by our new explant method, allowing us to obtain sufficient numbers of cells for more complete characterization. We report that T_RM_ cells are abundant in lung parenchyma, and these cells exhibit a distinct expression pattern of chemokine receptors on both CD4+ and CD8+ T cells. There was negligible contamination by peripheral blood T cells, since T_EM_ are rare in peripheral blood, no naïve T cells were recovered, and only small numbers of central memory phenotype T cells (T_CM_) were identified. Lung T_RM_ cells did not express CLA (skin homing) or α4β7 (gut homing) receptors, but did express high levels of VLA-1, also known as α1β1 integrin. With regard to T cell receptor phenotype, lung T_RM_ cells are also heterogeneous with regard to Vβ usage, in both CD4 and CD8 populations. Lung T_RM_ cells were not anergic, as judged by their capacity to produce cytokines rapidly upon activation. Many lung T_RM_ cells could be activated to produce TNFα, and IFNγ, while fewer expressed IL-17, IL-13 or IL-4 respectively. Most of the IFNγ secreting lung resident CD4+T cells co-expressed TNFα and IL-2, consistent with a “multifunctional Th1 type” phenotype. And finally, a significant subpopulation of lung T_RM_ cells proliferated in response to influenza antigen. In contrast, the influenza did not induce significant proliferation in populations of T cells from either blood or skin. This novel culture recovery method has enabled us to discover large numbers of functional and antigen specific T cell resident to human lung, suggesting that analogous to skin, immune memory for antigens encountered through the airways may reside in this novel population of T_RM_ cells.

## Methods

### Collection of Lung and skin biopsies

All scientific studies were approved by the Institutional Review Board (IRB) of the Partners Human Research Committee. Since de-identified pieces of lung, skin and gut tissue were acquired as discarded tissue for use in our studies, patient written or verbal consent was not required. As per our institute policy, IRB review is not required for research on (1) non-identifiable tissue or (2) coded tissue that is provided without linked identifiable information, when the tissue is obtained from IRB-approved Research Tissue Banks within Partners Healthcare. We obtained pieces of non-inflamed normal lung tissue from a site distant from a localized tumor from thirty patients of different ages and genders undergoing lung surgery for various types of malignancies localized to a specific segment of lung (Table S1).

### Isolation of T cells by conventional enzymatic methods

Lung tissue (1–2 gm) was minced in to very small pieces using scissors and kept in 2 ml tissue-digest mixture (0.2 µg/ml: Collagenase A, 40µg/ml: DNAse I, 1mM Ca++ in HBSS supplemented with 5% FCS, 10mM HEPES, penicillin and streptomycin and 3.5µl/L β-mercaptoethanol) for 2 h at 37°C. After incubation, enzyme activity was stopped by stop solution (20% FBS in HBSS) and passed through 70µm cell strainer. Cells were then resuspended in 1–2 ml PBS and layered on Ficoll-hypaque gradient mix and separated. Lymphocytes were recovered. Cell counts and phenotype was determined by BD Truecount tubes according to manufacturer's instruction and by flow cytometry.

### Isolation of T cells from lung, skin and gut using three-dimensional explant cultures

We isolated skin T cells by 3-dimensional explant method as described previously [5]. A similar method was used to isolate lung and gut T cells with the following modifications [5]. For the isolation of T cells, lung tissue (1–2 gm) or gut tissue (1–2 gm) was minced in to very small pieces using scissors and culture and minced tissue was loaded on the matrices (Statamatrix-TM matrices 9 mm diameter, pore width ∼500um, interconnecting pore width ∼80 to ∼200um, scaffold structure width ∼100um from Cytomatrix, Australia ). Matrices loaded with tissue were placed onto 24 well plate (1 matrix per well). T cells were harvested by aspiration of the culture medium and a thorough flushing of the matrices at different time points (where indicated). The culture was maintained in 2 ml/well IDMEM (Mediatech) with 10% AB serum (Lonza), Penicillin and streptomycin, and 3.5 µl/L β-ME. After 3 days, lung-T cells were harvested and used for further analysis. However, similar to skin, gut-T cells were harvested after 4-week of explant culture. For counting, cells were recovered from two matrices (2 wells) and total lymphocytes (CD45+), total T cells (CD3+), CD4+ T cells and CD8+ T cells were counted by BD Truecount tubes (BD Biosciences) and absolute numbers of each population were determined according to the manufacturer's instructions. The absolute numbers of cells in an adult normal human lung was calculated with following formula: (Absolute numbers of cells per matrix×total number of matrices /weight of tissue received).

### Enumeration of T cells in the lung

Identification of CD3, CD4 and CD8 and hematoxylin and eosin staining (H&E) was carried out on formalin fixed, paraffin embedded sections using standard routine method. T cells (CD3) present in extravascular connective tissue of alveolar septi were counted in sections 12 µm thick and 500 µm wide. We counted approximately 10,000 cells (9408±200 cells, n = 3) in 1 mm^3^ non-inflamed lung tissue.

### Flow cytometric analysis of T cells

Multi-color flow cytometric analysis of T cells was performed using directly conjugated monoclonal antibodies (mAbs). Anti-human CD1b (M-T101), CD3 (UCHT1) CD4 (SK3), CD8 (SK1), CD11c (B-ly6), CD14 (M5E2), CD19 (H1B19), CD25 (M-A251), CD45 (2D1), CD45RA (L48), CD45RO (UCHL1), CD56 (B159), CD69 (L78), invariant NK T cell (6B11), TCRαβ (T10B9.1A-31), TCRγδ (11F2), CCR3 (5E8), CCR4 (1G1), CCR5 (2D7/CCR5), CCR6 (11A9), CXCR3 (1C6/CXCR3) and CXCR4 (12G5), Cutaneous lymphocyte-associated Ag [(CLA), (clone HECA-452)] L-selectin (DREG-56), p-selectin glycoprotein ligand-1 [(PSGL-1), (clone: KPL-1)], HLA-DR (G46-6), and IL-1α (364-3B3-14), Abs were purchased from BD Biosciences Pharmingen (USA). The anti human CXCR6 (56811), CCR7 (150503), CCR8 (191704) and IL23R (218213) mAbs and recombinant human IL-2 and IL-15, were obtained from R&D Systems. FoxP3 staining set was purchased from ebiosciences (USA). Alpha4 beta7 (α4β7: ACT-1) was a gift from Dr. James J. Campbell (Brigham & Women's Hospital, Boston, USA).

For the detection of cytokine production, cells were cultured in Iscov/10%AB serum either with PMA (10 ng/ml) and ionomycin (500 ng/ml) for 6 h or TCR stimulation by microbeads coated with anti CD2, anti CD3 and anti-CD28 (from miltenyi biotech) for overnight (approximately 16 h) in presence of brefeldin A (BD). After incubation time, cells were stained for surface receptor, fixed & permeabilized (Cytofix/cytoperm kit: BD Pharmingen) and stained for intracellular cytokines [anti human IL-13 (JES10-5A2) and IL-4 (8D4-8) were purchased from BD pharmingen. The fast immune anti human TNFα (6401.1111), IL-1β (AS10) and IFNγ (25723.11) were purchased from Becton-Dickinson. The anti human IL-17 (eBio64DEC17) was purchased from eBiosciences]. Analysis of flow cytometry samples was performed on a BD Biosciences FACS Canto instrument, and data were analyzed with FACS Diva software.

### Ki67 staining

Cells from lung T cell explant culture, PBMCs and KG1a cells were harvested. Cells were pellet down by centrifugation (1200 rpm for 5 min) and 70% ethanol was added dropwise into the cell pellet (1–5×10^7^ cells) followed by incubation (−20° for 2 hours). After incubation, cells were washed twice with 30 ml of staining buffer (PBS with 1% FBS, 0.09% NaN_3_). Cells (1×10^7^ cells/ml) were resuspended in staining buffer and 100µl cells suspension (1×10^6^ cells) were transferred into each sample tube. PE labeled Anti-Ki67 antibody (20µl/sample: Clone B56, Cat No. 556003 from BD pharmingen, USA) or isotype was added into the tubes. Cells were incubated for another 20–30 min in the dark at room temperature. After incubation, 0.5ml staining buffer was added into each tube. Before flow cytometric analysis, 20µl BD Via-probe™ solution was added to exclude dead cells.

## Results

### Non-inflamed human lung contains abundant T cells

**Figure 1:**
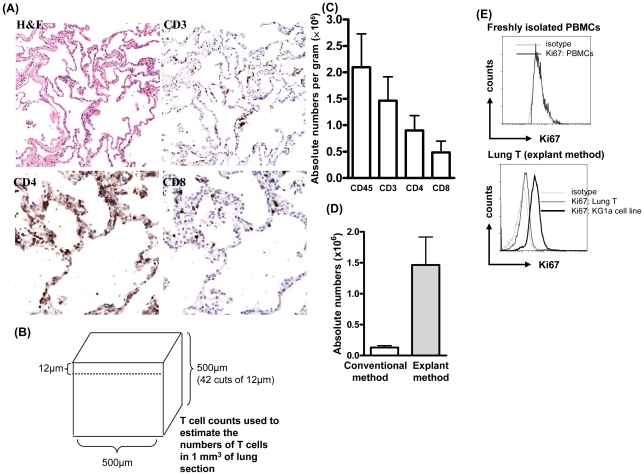
Human lung contains large numbers of T cells. (A) Non-inflamed normal lung tissue was obtained and stained for hematoxylin and eosin (H&E), CD3 (200×), CD4 and CD8 (400×). A representative experiment is shown, and three additional donors produced similar results. (B) T cells were counted in sections 12 µm thick and 500 µm wide and estimated the numbers of T cells in 1 mm^3^. (C) After 3-day of explant culture, lung-T cells were harvested and counted as described in Materials & Methods. Data is shown as Mean ± SEM of 10 experiments. (D) Lung-T cells were extracted from same specimen by conventional as well as lung explant method and counted as described in Materials and Method section. Data is shown as Mean ± SEM of 3 experiments. (E) Lung-T cells, Blood T cells from freshly isolated PBMCs (negative control), or KG1a cells (positive control) were stained for Ki67 and analyzed by flow cytometry.

We obtained human lung tissue from patients undergoing partial or complete pneumonectomy for localized tumor with intent to cure. Only tissue judged to be 1) at a significant distance from the tumor, and 2) that was grossly normal in appearance, was released by the BWH Department of Pathology as “discarded tissue” for use in our studies. We defined “non-inflamed normal” lung by the following criteria: (1) no gross clinical evidence of any inflammation. (2) histological examination of obtained tissue by H&E showed no signs of emphysema, fibrosis, intra-alveolar transudate (edema), intra-alveolar exudate (pneumonia) or any known infection. By these criteria, the lung tissue used for our analyses was non-inflamed and histologically normal. By immunohistochemical analysis (Figure 1A), the majority of T cells are present in extravascular connective tissue of alveolar septi, and these CD3+ T cells include CD4+ as well as CD8+ T cells. Figure 1A reinforces the observation that histologically, these segments of lung were indistinguishable from lung obtained at autopsy in patients who did not die of lung disease (Figure S1).

We counted the T cells directly in fixed and immunostained sections of normal lung (Figure 1A–B). T cells (CD3) present in extravascular connective tissue of alveolar septi were counted in sections 12 µm thick and 500 µm wide (Figure 1B). We counted approximately 10,000 cells (9408±200 cells, n = 3) in 1 mm^3^ non-inflamed lung tissue. Extrapolating from this, there are roughly 10^7^ T cells per cubic centimeter (cc), or 10^10^ T cells (10 billion) in an adult human lung (weight of adult human lung: 1000gm). This is comparable to the total number of T cells in human blood.

### Isolation of large numbers of non-expanded T_RM_ cells from human lung

Previous studies examining T cells in human lung have reported that relatively few cells could be extracted by conventional methods [13], [14], [15], [16], [17]. As a result, only limited phenotypic analysis could be performed, and functional significance of lung T_RM_ cells in pulmonary disorders remains unclear. T cells were isolated from lung parenchyma using either a conventional enzymatic method or by explant culture with Statamatrices as described in *Materials &* *Methods* section (4). Cells were harvested from explant culture after 3 days and lymphocytes (CD45) and T cells (CD3, CD4 and CD8) were counted (Figure 1C). The majority of cells (>90%) in the CD45+ population were CD3+ T cells. Among the CD3+ T cells, the relative numbers of CD4+ T cells (Mean ± SEM: 55±5%, range: 30–76%) and CD8+ T cells (Mean ± SEM: 39±5%, range: 18–66%) were variable between donors, but there was a trend towards higher numbers of CD4+ T cells (n = 6 out of 8 individuals) as compared with CD8+ T cells. By our explant method, we determined that one gram of lung tissue yielded large numbers of CD3+ T cells (1.5×10^6^), CD4+ T cells (1×10^6^) and CD8+ T cells (0.5×10^6^) (Figure 1C). Considering the average weight of human lung as 1000 gm, we estimated that roughly 1.5 billion CD3+ T cells (1.5×10^9^) can be extracted by explant method. However, based on our counting of sections, it appears that our efficiency of isolating T cells from lung may be as low as 15–20%. Using lung explant method, approximately 10 times more T cells (up to 2 million) were extracted as compared with conventional method (up to 0.2 million) from one gram of lung tissue (Figure 1D). T cells isolated from lung did not spontaneously proliferate in vitro. Ki67 expression was analyzed. Lung T cells and freshly isolated PBMCs showed negligible Ki67 staining; however KG1a (human myeloid leukemia cells) cultured in similar conditions showed bright Ki67 staining (Figure 1E).

### Direct comparison of conventional enzymatic methods and lung matrix explant methods

**Figure 2:**
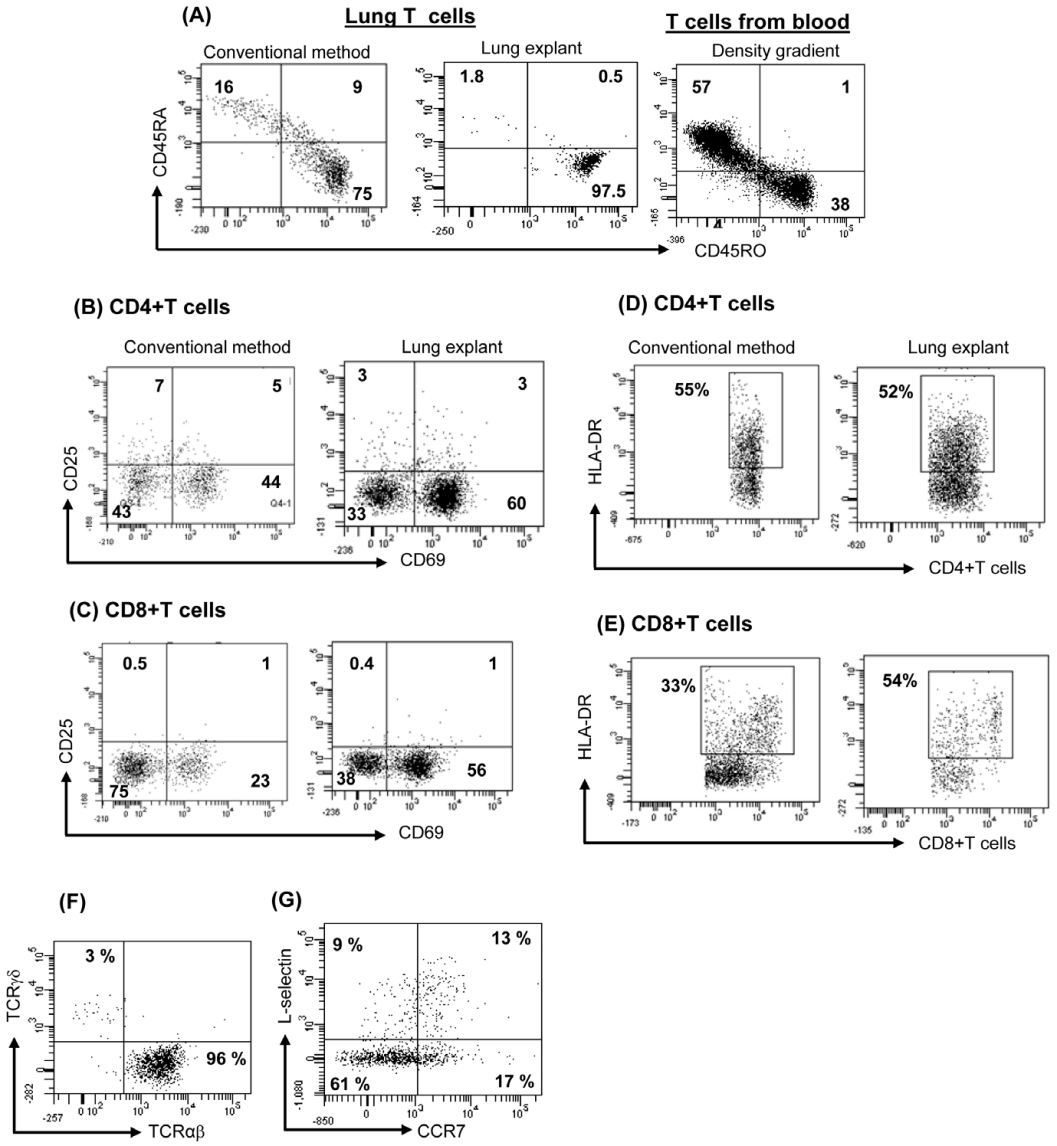
Direct comparison of conventional enzymatic methods and lung matrix explant methods. T cells were extracted by conventional method and explant method. PBMCs were isolated by Ficoll-hypaque density gradient method. Cells were stained for surface markers and analyzed by flow cytometry. (A) Expression of CD45RO (T_EM_) and CD45RA on lung-CD3+T cells and blood-T cells was analyzed. (B–E) Many CD4 and CD8+T cells express CD69, HLA-DR and only few CD4+T cells but not CD8 express CD25. (F) Most of the lung-T cells are αβ+ and very few express γδ TCR. (G) A subpopulation of CD4+ CD45RA- T cells also express L-selectin and CCR7 (markers for central memory phenotype). Dot plots are representative of 9 experiments (3A–F) that produced similar results.

We next characterized the phenotype of T cells extracted from same lung specimen by both methods. Using lung explant culture, the majority of T cells express CD45RO (>90%), a marker for memory phenotype and very few (<5%) express CD45RA (naïve cells) (Figure 2A). A somewhat higher percentage of CD45RA+ T cells (15–25%) were present if T cells were extracted by conventional method. This is evidence that T cells extracted by the lung explant methods had minimal contamination with peripheral blood T cells, which are typically >50% CD45RA+ (Figure 2A). Expression of various activation markers (CD25, CD69 and HLA-DR) was not different on CD4+T cells isolated from different methods, but CD8+T cells extracted from lung explant showed higher expression of CD69 and HLA-DR as compared with conventional method (Figure 2B–E).The majority of CD3+ T cells were TCRαβ+ (>95%) and few were TCRγδ+ T cells (2–5%) (Figure 2F). A subset of CD4+CD45RA− population co-expresses CCR7 and CD62L, suggesting the presence of T_CM_ in the lung (Figure 2G). A subpopulation of CD4+ T cells expressing CD25 and FoxP3 was identified as well (CD4+CD25+FoxP3+ T cells: 1.5%±1%, n = 6), suggesting the presence of T_reg_ cells.

We wished to be certain that the T cells we were isolating from lung reflected a population resident to lung, rather than peripheral blood T cells trapped in pulmonary capillaries. If the latter were true, we would expect to see roughly 50% of both CD4+ and CD8+ T cells expressing CD45RA, L selectin, CCR7, and low levels of α4β7, all markers of naïve T cells in peripheral blood. In fact, virtually all of the lung resident T cells were CD45RO+, negative for CD45RA and α4β7, and few expressed either L selectin or CCR7 (Figure 2A and Figure 2G). Since the vast majority of CD45RO+ cells in peripheral blood also co-express L selectin and CCR7 (i.e., central memory cells), and only a minority have an effector memory phenotype (lacking CCR7 and L selectin), we conclude that the cells we have isolated from lung are not representative of peripheral blood T cells, and thus do not represent contamination with intravascular cells trapped in pulmonary capillaries. Careful examination of sections of lung stained with immunoperoxidase conjugated antibodies to T cell markers confirmed the location of these cells in the extravascular space. Thus, the cells we have studied represent an authentic tissue resident population.

### Lung T_RM_ cells express VLA-1 (α1β1)

**Figure 3:**
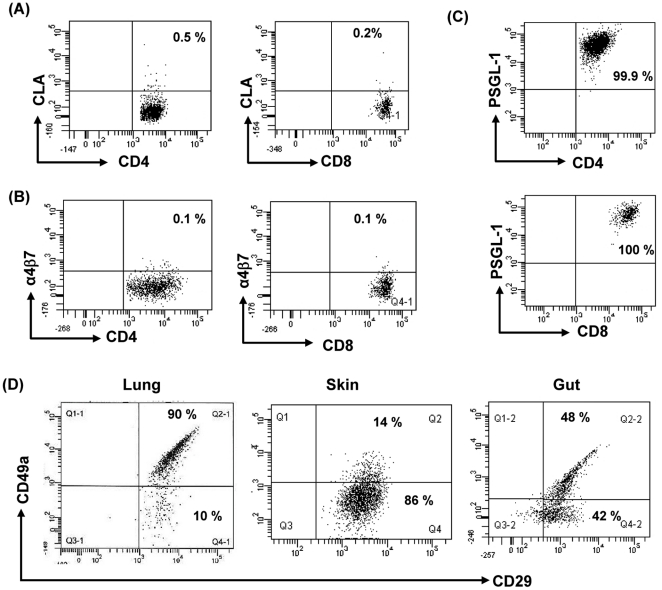
Lung T cells uniformly express VLA-1 and PSGL1 but not skin homing (CLA) and gut homing (α4β7) markers. Lung T cells were extracted by lung explant method and stained for CLA (HECA-452) (A), α4β7 (ACT-1) (B), and PSGL-1 (C) and analyzed by flow cytometry. Lung-T cells, skin-T cells and gut-T cells were stained for VLA-1 (CD49a+CD29+) and analyzed by flow cytometry (D). A representative dot plot of each marker is shown and 3 experiments (α4β7, PSGL-1, VLA-1) and 2 experiments (CLA) produced similar results.

Similar to previous reports [13], [16], [18], lung-T cells extracted by lung explant method express negligible CCR3, CCR8 and CXCR6, and expression of CCR4 on CD4+ T (∼25%) and CD8+ T cells (∼5%) was modest. Most of the lung CD4+ T cells expressed CCR5, CCR6, CXCR3, and CXCR4. CD8+ T cells also express similar level of these receptors with the exception of CCR6 (Table 1). Lung T cells uniformly express the β1 integrin VLA-1 and the addressin PSGL-1, but not CLA (skin homing) or α4β7 (gut homing) (Figure 3A–C). VLA-1 (CD49a+CD29+) expression seems to be relatively specific for lung T cells; only 14% of skin resident T cells, and 48% gut resident T cells, express VLA-1 (Figure 3D).

**Table 1:** Expression of chemokine receptors on lung T_RM_ cells.

	CD4+ T cells(%)	CD8+ T cells (%)
**CCR4**	24±3	5±1
**CCR5**	63±3	58±3
**CCR6**	61±4	29±4
**CXCR3**	82±3	92±2
**CXCR4**	81±4	80±4
**VLA-1**	75±5	90±3

Frequency (in %age) of CD4+T cells and CD8+T cells expressing indicated receptors are shown as Mean ± SEM from 4 experiments.

### Cytokine Production by Lung T_RM_ Cells

**Figure 4:**
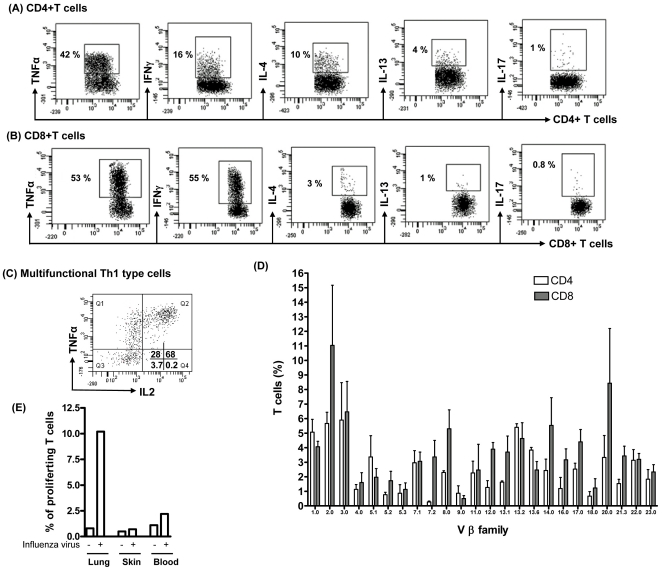
Large numbers of immunocompetent and influenza-specific T cells resides in human lung. Lung T cells were extracted by lung explant method. (A–B) The cytokine secretion of effector memory T cells after overnight stimulation with artificial APCs (microbeads coated with anti-CD2, anti CD3 and antiCD28 mAbs) at 1∶1 cells: bead ratio. Brefeldin A (golgi-stop) was added 6 h prior to intracellular staining of cytokines. A representative dot plot of each cytokine is shown and 6 additional experiments produced similar results. (C) CD4+T cells were stained with TNFα, IL-2 and IFNγ after stimulation with PMA+ionomycin for 6h in presence of brefeldin A (Gate on CD4+IFNγ+ population). A representative dot plot is shown and 10 additional experiments produced similar results. (D) Lung T cells were isolated and stained for different Vbeta T cell receptors using TCR V beta repertoir kit (Beckman coulter) according to manufacturer's instructions. Diversity of V beta TCRs was analyzed by flow cytometry. Data represent Mean +/− SEM of 3 different donors. (E) CFSE labeled T cells from lung, skin and blood were cultured with heat killed influenza virus pulsed APCs in 1∶2 ratio. On day 4, T cell proliferation was measured by analyzing CFSE dilution using flow cytometry. A representative experiment is shown and 2 additional experiments produced similar results.

Previous studies suggested that T cells resident to lung parenchyma may be anergic [19]. We therefore asked if lung resident T cells were functional, as judged by cytokine production. Since lung resident T cells are largely of the T_EM_ phenotype, we investigated their capacity to express cytokines. While there was negligible baseline secretion of cytokines (Figure S2), CD4+T cells and CD8+T cells treated with artificial APCs (microbeads coated with anti-CD2, anti-CD3 anti-CD28 mAbs) in presence of brefeldin-A produced TNFα, IFNγ, IL-4, IL-13 and IL-17. A representative experiment depicts that the majority of the T cells stimulated with artificial APCs secreted IFNγ while relatively fewer cells also secreted IL-4, IL-13 and IL-17 (Figure 4A–B). IFNγ secreting CD4+T cells that express TNFα and IL2 have been defined as “multifunctional” Th1 type cells, and are thought to be associated with superior host defense [20]. We therefore asked if lung CD4+ T_RM_ cells were multifunctional with regards to cytokine production. Figure 4C shows that IFNγ secreting CD4+ T_RM_ cells can co-express TNFα and IL-2, a characteristic feature of multifunctional Th1 type cells. We conclude that the majority of T cells isolated from normal human lung can produce at least one cytokine, indicating that these cells are not anergic.

### Antigen Specificity of Lung T_RM_ Cells

We analyzed Vβ segments of TCR using a TCR Vβ repertoire kit to understand the diversity of TCR repertoire in lung resident T cells. Figure 4D depicts that lung T cells (CD4 and CD8 T cells) have a highly diverse TCR repertoire thus lung contains a very complex T cells population rather than few dominant clones.

In order to determine whether lung contains functional T_RM_ cells enriched for those specific for antigens encountered through airways, we extracted lung T cells, skin T cells and blood T cells (from PBMCs). CFSE labeled lung T cells, skin T_RM_ cells and blood T cells were cultured with APCs (non-T cells mononuclear cells from lung, skin and PBMCs respectively) pulsed with heat killed influenza virus (B/Allen/45:VR-102 from ATCC, USA). For comparison, CFSE loaded skin T cells and blood T cells (PBMCs) were also cultured with influenza pulsed APCs as controls. After 4 days, lung T cell, skin T_RM_ cells and blood T cell proliferation was analyzed by assessing CFSE dilution using flow cytometry. Strikingly, significant numbers of lung T_RM_ (14%), but very few blood T cells (1%) and no skin T_RM_, proliferated in response to influenza (Figure 4E & Figure S3). This suggests that lung T_RM_ are enriched for T cells specific for antigens like influenza virus that are typically encountered through respiratory mucosa. In addition, these data emphasized that lung T_RM_ were not contaminated with blood T cells. If later is true, we would expect the similar numbers of influenza specific T cells in lung and blood.

## Discussion

In this study, we demonstrate that human lung contains large numbers of resident memory T cells (T_RM_;) there are roughly as many T cells resident in human lung as in peripheral blood. These T_RM_ cells have a distinct cell surface phenotype, are diverse with regard to T cell repertoire, produce multiple cytokines, and are specific for antigens previously encountered via the respiratory route. The vast majority of T_RM_ cells in human lung have the phenotype of T_EM_, which is the identical memory phenotype of skin T_RM_ T cells. These data strongly suggest that lung contains a population of memory T cells that can respond rapidly to challenges from the environment.

In this study, we obtained normal-appearing sections of human lung intraoperatively from patients undergoing pneumonectomy for cancer. The normal appearing parts of the resected lung were grossly and microscopically indistinguishable from normal lung obtained at autopsy. Immunohistochemical analysis of normal-appearing lung revealed the presence of both CD4 and CD8 positive T cells, and no evidence of acute inflammation or infection. Counting of CD3 positive cells revealed large numbers of T cell present. Immediately after harvest, a method originally developed to extract viable T cells from human skin in our lab was applied to human lung (13). Approximately 10 times more T cells were extracted as compared with conventional method. Numerous experiments in more than 25 separate donors yielded similar results. Extraction of T cells from skin [4] and lung (Figure 2) by explant method did not alter the phenotype of T cells (expression of various surface molecules CLA, CD25, CD69 etc) compared to conventional method. Within this population, CD4+ T_RM_ cells outnumbered CD8+ T_RM_ cells, and virtually all T_RM_ cells analyzed were CD45RO+ and bore αβ T cell receptors. Only a small minority of these T_RM_ cells co-expressed CCR7 and L selection, confirming that the majority of T_RM_ cells were, at least phenotypically, T effector memory cells (T_EM_). Importantly, no naïve T cells, and only a small fraction of central memory cells (T_CM_), could be identified, effectively ruling out the possibility that the cells we extracted were simply peripheral blood cells trapped in pulmonary capillaries.

Similar to T cells extracted from other peripheral tissues [4], lung T_RM_ cells expressed the activation marker CD69. A significant population of T_RM_ cells expressed HLA-DR, and a minority of CD4 T cells co-expressed CD25 and FoxP3, marking them as putative T regulatory cells [21]. The α1 integrin VLA-1 was expressed on virtually all lungT_RM_ cells; in contrast, it was present on only 30% of skin T_RM_ T cells, and an even smaller percentage of gut T_RM_ cells. VLA-1, or α1β1 is a receptor for type IV collagen, and the significance of its abundance in lung T_RM_ cells is unknown. With regard to tissue homing markers, neither CLA (skin) nor α4β7 (gut) were expressed by lung T_RM_ cells, though all expressed the protein backbone for the CLA tetrasaccharide (PSGL-1) [22], [23], [24]. Similarly, the pattern of chemokine receptor expression on lung resident T cells was different from T cells isolated from either skin or GI tract, with abundant CCR5, CXCR4, and CCR6 being expressed.

Lung T_RM_ cells contain a diverse TCR repertoire. These data suggest that lung contains a very large repertoire of immunocompetent T_RM_ cells and lung T_RM_ cells may be poised at the environmental interface to respond to pathogen encountered in that setting. One of the most striking finding of this study was to observe the large numbers of immunocompetent T cells, including influenza specific T_RM_ cells in the lung. A majority of lung T_RM_ cells expressed at least one cytokine upon activation indicating that these cells were not anergic, and were capable of effector cytokine production. In fact, a significant number of CD4+ T_RM_ cells co-expressed IL-2, TNFα, and IFNγ upon activation, marking them as so-called multifunctional Th1 type cells [20]. The abundance of multifunctional T_RM_ cells was actually higher than what has been observed in both blood and skin (not shown). Interestingly, significant numbers of influenza specific T_RM_ cells were observed in the lung. However, blood contains very few influenza specific T cells and there were no influenza specific T_RM_ cells detected in the skin.

These results support the increasingly popular idea that peripheral tissues actually contain large numbers of functional T_RM_ cells that are enriched for T cells specific for antigens encountered through that tissue's interface with the environment. This suggests tissue T_RM_ cells are poised to maintain the immune system's most distal defenses, and suggests that acute recruitment of memory T cells from blood or lymph node may not be necessary to initiate a recall immune response in skin, lung or GI tract. Understanding the natural history of these tissue T_RM_ cells is likely to shed considerable light upon physiologic host defense, as well as the pathophysiology of T cell mediated diseases like asthma, psoriasis, and inflammatory bowel disease.

## Supporting Information

Figure S1 **Human lung contains large numbers of T_RM_.** Lung obtained at autopsy in a patient who did not die of lung disease was stained for hematoxylin and eosin (H&E), and CD3.
(TIF)

Figure S2 **Cytokine expression by lung T_RM_.** The cytokine secretion of effector memory T cells at baseline level was analyzed by intracellular cytokine staining. A representative dot plot of each cytokine is shown and 6 additional experiments produced similar results.
(TIF)

Figure S3 **Influenza specific T_RM_ resides in human lung.** CFSE labeled T cells from lung, skin and blood were cultured with heat killed influenza virus pulsed APCs in 1∶2 ratio. On day 4, T cell proliferation was measured by analyzing CFSE dilution using flow cytometry. A representative dot plot of each group is shown and 2 additional experiments produced similar results.
(TIF)

Table S1 (DOC)
