# Supplementary figures and images for: Resident Memory T Cells (TRM) Are Abundant in Human Lung: Diversity, Function, and Antigen Specificity
Source: PLoS One. 2011 Jan 26;6(1):e16245. doi: 10.1371/journal.pone.0016245 (PMC3027667; doi:10.1371/journal.pone.0016245)

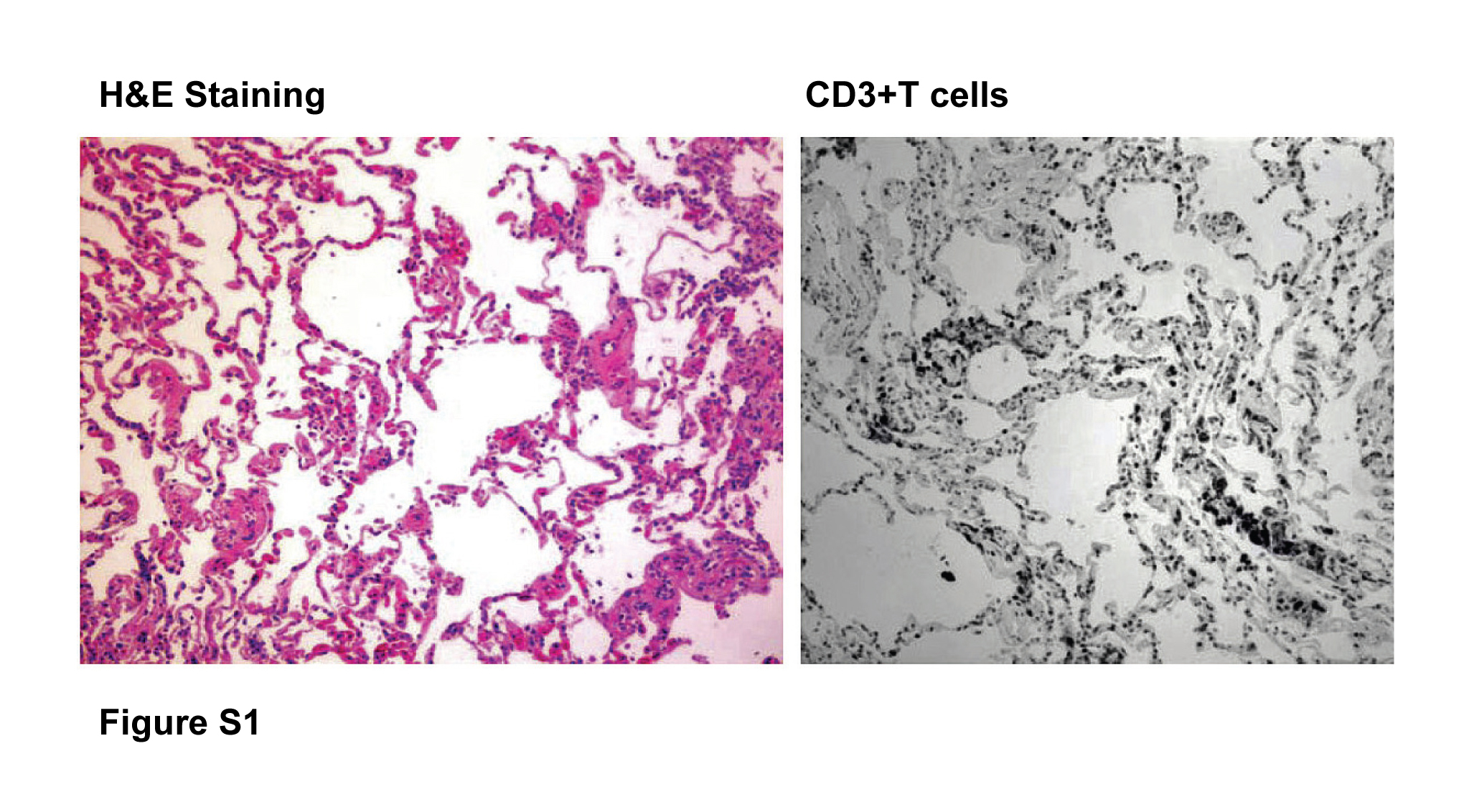

Supplement: Figure S1 — Human lung contains large numbers of TRM. Lung obtained at autopsy in a patient who did not die of lung disease was stained for hematoxylin and eosin (H&E), and CD3. (TIF) [file pone.0016245.s001.tif]

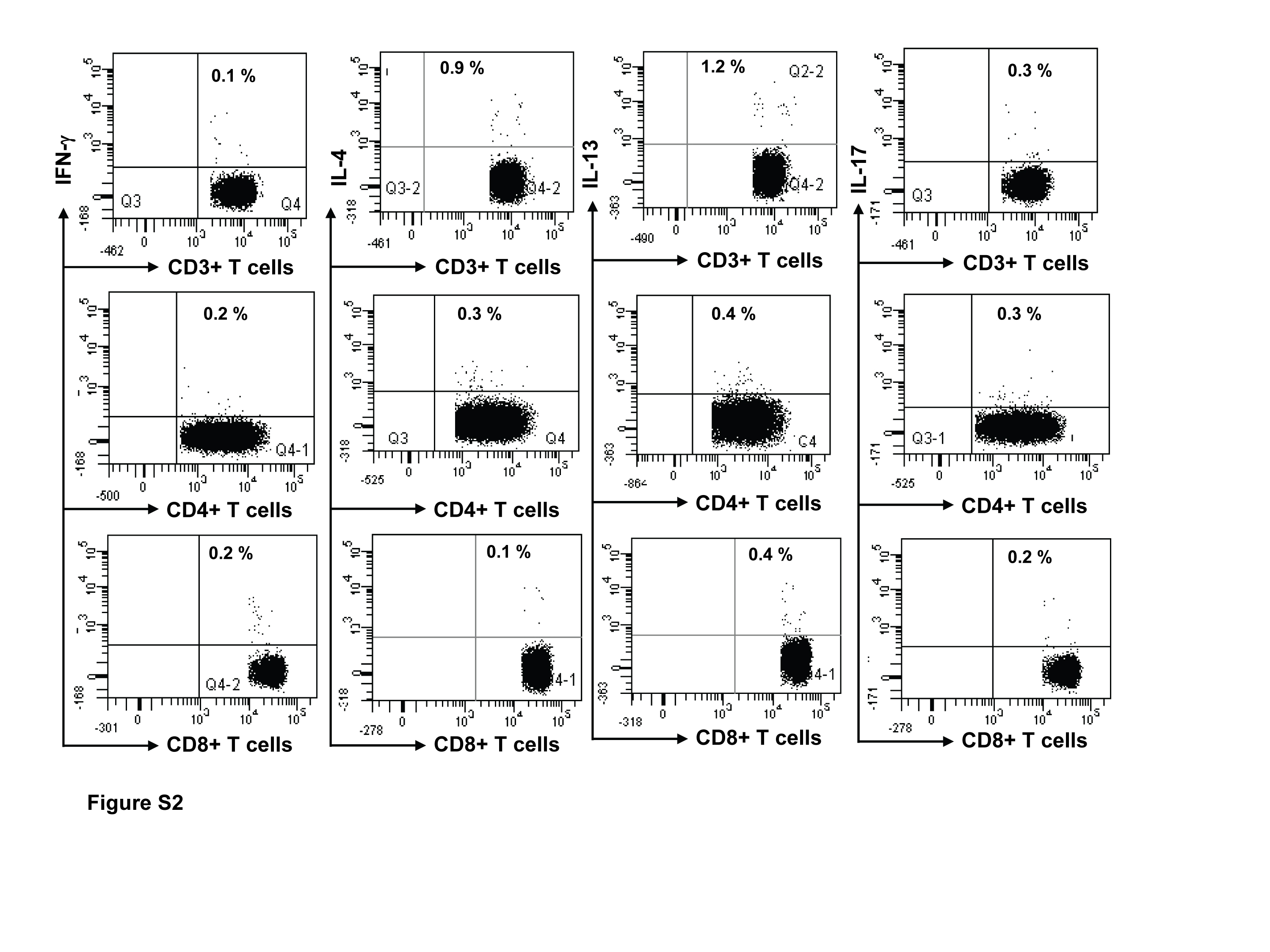

Supplement: Figure S2 — Cytokine expression by lung TRM. The cytokine secretion of effector memory T cells at baseline level was analyzed by intracellular cytokine staining. A representative dot plot of each cytokine is shown and 6 additional experiments produced similar results. (TIF) [file pone.0016245.s002.tif]

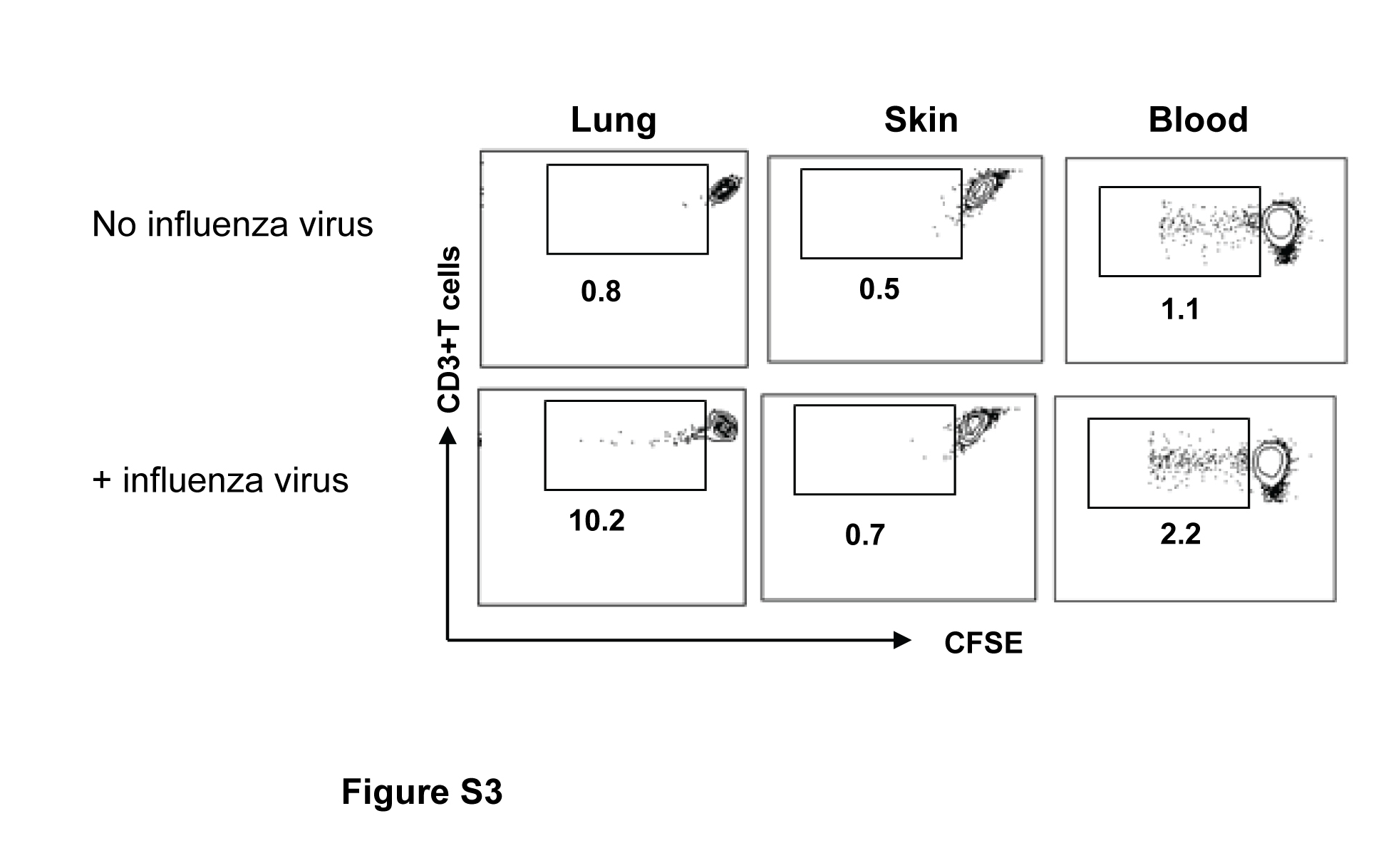

Supplement: Figure S3 — Influenza specific TRM resides in human lung. CFSE labeled T cells from lung, skin and blood were cultured with heat killed influenza virus pulsed APCs in 1∶2 ratio. On day 4, T cell proliferation was measured by analyzing CFSE dilution using flow cytometry. A representative dot plot of each group is shown and 2 additional experiments produced similar results. (TIF) [file pone.0016245.s003.tif]
