# Supplementary material for: Resident Memory T Cells (TRM) Are Abundant in Human Lung: Diversity, Function, and Antigen Specificity
Source: PLoS One. 2011 Jan 26;6(1):e16245. doi: 10.1371/journal.pone.0016245 (PMC3027667; doi:10.1371/journal.pone.0016245)
Supplement: Table S1 — (DOC) [file pone.0016245.s004.doc]

|  | **Characterization of specimen** |
| --- | --- |
| Number of donors | 30 |
| Age (years) | 47-87 |
| Gender  Male Female | 20  10 |
| Indication for surgery | localized tumor |

**Table S1**

Details of individuals recruited in the study are described.
